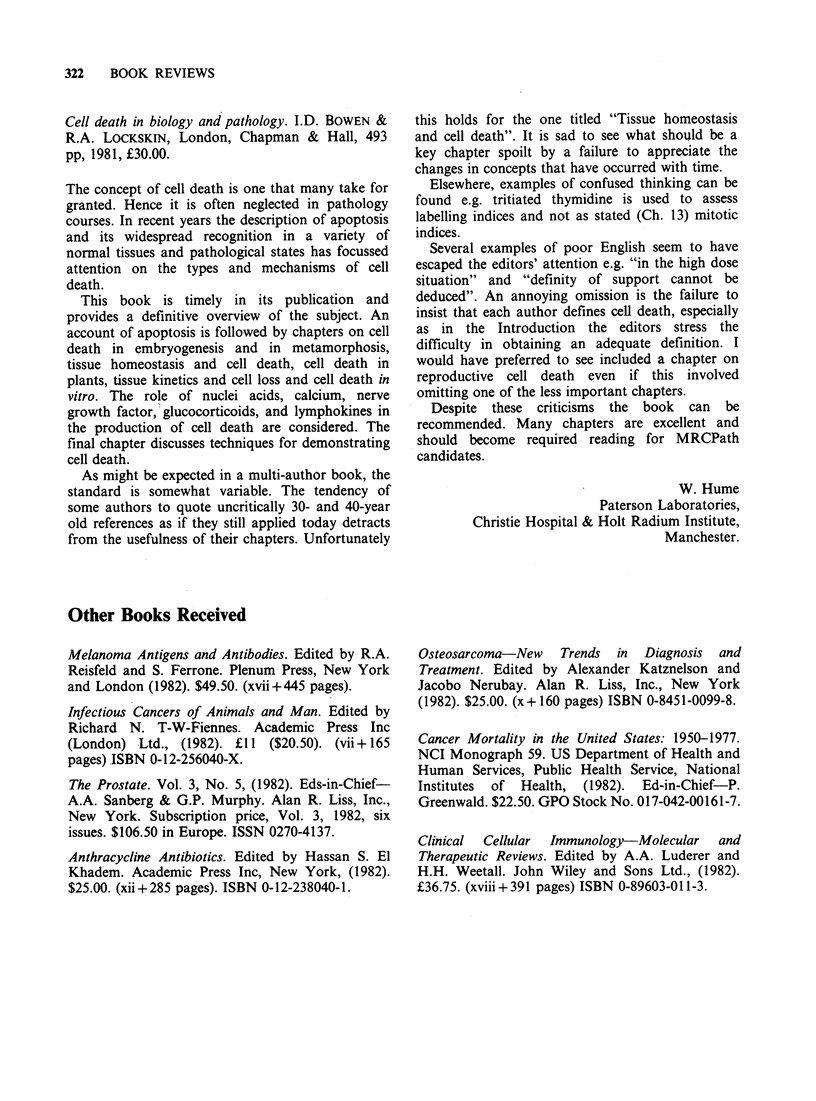# Cell death in biology and pathology

**Published:** 1983-02

**Authors:** W. Hume


*Cell death in biology and pathology*. I.D. BOWEN & R.A. LOCKSKIN, London, Chapman & Hall, 493 pp, 1981, £30.00.

The concept of cell death is one that many take for granted. Hence it is often neglected in pathology courses. In recent years the description of apoptosis and its widespread recognition in a variety of normal tissues and pathological states has focussed attention on the types and mechanisms of cell death.

This book is timely in its publication and provides a definitive overview of the subject. An account of apoptosis is followed by chapters on cell death in embryogenesis and in metamorphosis, tissue homeostasis and cell death, cell death in plants, tissue kinetics and cell loss and cell death *in vitro*. The role of nuclei acids, calcium, nerve growth factor, glucocorticoids, and lymphokines in the production of cell death are considered. The final chapter discusses techniques for demonstrating cell death.

As might be expected in a multi-author book, the standard is somewhat variable. The tendency of some authors to quote uncritically 30- and 40-year old references as if they still applied today detracts from the usefulness of their chapters. Unfortunately this holds for the one titled "Tissue homeostasis and cell death". It is sad to see what should be a key chapter spoilt by a failure to appreciate the changes in concepts that have occurred with time.

Elsewhere, examples of confused thinking can be found e.g. tritiated thymidine is used to assess labelling indices and not as stated (Ch. 13) mitotic indices.

Several examples of poor English seem to have escaped the editors' attention e.g. "in the high dose situation" and "definity of support cannot be deduced". An annoying omission is the failure to insist that each author defines cell death, especially as in the Introduction the editors stress the difficulty in obtaining an adequate definition. I would have preferred to see included a chapter on reproductive cell death even if this involved omitting one of the less important chapters.

Despite these criticisms the book can be recommended. Many chapters are excellent and should become required reading for MRCPath candidates.

W. Hume
Paterson Laboratories,
Christie Hospital & Holt Radium Institute,
Manchester.